# IL-17 Signaling in Primary Sclerosing Cholangitis Patient-Derived Organoids

**DOI:** 10.21203/rs.3.rs-3406046/v1

**Published:** 2023-10-16

**Authors:** Ana Sofia Garcia Moreno, Maria Eugenia Guicciardi, Alexander Q. Wixom, Erik Jessen, Jingchun Yang, Sumera I. Ilyas, Jackie K. Bianchi, Filippo Pinto e Vairo, Konstantinos N. Lazaridis, Gregory J. Gores

**Affiliations:** Mayo Clinic, Rochester, MN, USA; Mayo Clinic, Rochester, MN, USA; Mayo Clinic, Rochester, MN, USA; Mayo Clinic, Rochester, MN, USA; Mayo Clinic, Rochester, MN, USA; Mayo Clinic, Rochester, MN, USA; Mayo Clinic, Rochester, MN, USA; Mayo Clinic, Rochester, MN, USA; Mayo Clinic, Rochester, MN, USA; Mayo Clinic, Rochester, MN, USA

## Abstract

The pathogenesis of primary sclerosing cholangitis (PSC) is unclear, although studies implicate IL-17A as an inflammatory mediator in this disease. However, a direct assessment of IL-17 signaling in PSC cholangiocytes is lacking. In this study we aimed to investigate the response of PSC extrahepatic cholangiocyte organoids (ECO) to IL-17A stimulation. Cholangiocytes obtained from PSC and non-PSC patients by endoscopic retrograde cholangiography (ERC) were cultured as ECO. The ECO were treated with vehicle or IL-17A and assessed by transcriptomics, secretome analysis, and genome sequencing (GS). Unsupervised clustering of all integrated scRNA-seq data identified 8 cholangiocyte clusters which did not differ between PSC and non-PSC ECO. However, PSC ECO cells demonstrated a robust response to IL-17 treatment, noted by an increased number of differentially expressed genes (DEG) by transcriptomics, and more abundant chemokine and cytokine expression and secretion. After rigorous filtering, GS identified candidate somatic variants shared among PSC ECO from unrelated individuals. However, no candidate rare variants in genes regulating the IL-17 pathway were identified, but rare variants regulating the MAPK signaling pathway were present in all PSC ECO. In conclusion, PSC and non-PSC patient derived ECO respond differently to IL-17 stimulation implicating this pathway in the pathogenesis of PSC.

## Introduction

Primary sclerosing cholangitis (PSC) is a heterogeneous, chronic cholestatic liver disease characterized by fibro-inflammatory biliary tract strictures of the intra- and extrahepatic bile ducts. The resultant fibro-inflammatory process is usually progressive, and patients with PSC frequently advance to end-stage liver disease necessitating liver transplantation to extend survival.^[1]^ Rational therapies for PSC are lacking and will require an understanding of its molecular and cellular pathogenesis, especially insight into the mechanisms causing persistent injury to cholangiocytes (the cells lining the bile ducts). Although regarded as an immune-mediated disease, the pathogenesis of PSC remains elusive, mainly due to difficulty in accessing cholangiocytes, the instability of in vitro monolayer culture systems of primary cells and the lack of animal models which reproducibly recapitulate the human disease.^[2, 3]^ Over the last few years, a new cell-based model has been developed to address several of these deficiencies termed three dimensional (3D) organoid culture systems. Cholangiocyte organoids permit primary human cells to self-organize through cell-cell and cell-matrix interactions and have proven to be a powerful tool in the study of development, disease pathogenesis, and regeneration of the liver.^[4, 5]^ Primary cholangiocytes have been successfully obtained from bile and/or brushing collected during clinically indicated endoscopic retrograde cholangiography (ERC) procedures and grown into organoids.^[2, 6, 7, 8]^ In PSC, these patient-derived cholangiocyte organoids retain immunoreactive characteristics associated with PSC and can be maintained long term *in vitro*.^[2]^ Due to regional diversity within the human biliary tree it is important to obtain cells from large bile ducts, which are usually involved in PSC.^[9, 10]^ The availability of human disease-derived extrahepatic cholangiocyte organoids (termed ECO by consensus)^[5]^, permits the interrogation study of disease alterations afflicting cholangiocytes in PSC, such as those caused by inflammatory cytokines.

The role of the interleukin 17 (IL-17) signaling pathway has been described in several diseases, including those with an immune etiology, and this pathway has been successfully targeted therapeutically in human diseases such as psoriasis and ankylosing spondylitis.^[11, 12]^ The IL-17 family of ligands consists of six members, IL-17A to IL-17F, and its respective 5 member receptor family (IL17RA-IL17RE).^[13]^ IL-17A is prominently expressed in human diseases and has been broadly studied, therefore it is considered to be the main inducer of the IL-17 signaling pathway.^[14, 15]^ It shares a great percentage of conservation with IL-17F and it is produced predominantly by a T-cell subset termed Th17 cells.^[15, 16]^ IL-17A can be secreted by other cell types, including γδ T-cells, mucosal-associated invariant T (MAIT) cells, CD8^+^ T-cells and neutrophils in the liver. ^[17, 18, 19]^ Th17 cells and IL-17^+^ CD8^+^ T-cells are considered, however, the major source of IL-17 in several inflammatory liver diseases.^[20]^ In PSC, previous human studies have reported that IL-17-producing cells aggregate within periductal areas and that peripheral blood mononuclear cells in PSC patients induce a high Th17 cell response to pathogens *in vitro*.^[21, 22]^ Correspondingly, *Mdr2*^−/−^ and bile duct ligated mice, both models of cholestatic liver injury, demonstrate an increase in expression of hepatic IL-17A and its receptor IL-17RA, and aggregation of IL-17 producing cells in periductal areas.^[23, 24]^ In addition, other studies have reported a decrease in hepatic neutrophil accumulation, liver fibrosis, and liver damage in IL-17A or IL-17RA knockout mice or by blocking IL-17A. ^[24, 25, 26, 27, 28]^ These studies suggest an injurious role for IL-17 signaling in cholangiocytes and cholestatic liver injury. However, a recent study in mice cholangiocyte organoids determined that IL-17 induces programmed cell death ligand-1 (PD-L1) expression in cholangiocytes;^[29]^ induction of PD-L1 would be expected to impair T cell activation and limit T cell-mediated liver injury. Hence, the role of IL-17A in cholestatic liver injury is complex and requires further definition.

Recently, a study involving patients with concomitant PSC and inflammatory bowel disease (IBD), reported a transcriptional signature associated with an increased risk of colon dysplasia that is characterized by a pathogenic IL-17 signature in T cells^[30]^. In addition, studies conducted on colon organoids from patients with IBD identified somatic mutations, which dysregulate the IL-17 signaling pathway.^[31]^ These observations are quite pertinent to PSC, as the majority of PSC patients have IBD, and parallels exist in the mucosal injury of the colon and bile ducts in these diseases. However, information regarding somatic mutations and/or dysregulated IL-17 signaling in PSC cholangiocytes is lacking. Therefore, our aims were to characterize the response of PSC derived ECO to IL-17, with the goal of determining if IL-17 signaling is differentially regulated in PSC vs. non-PSC cholangiocytes.

## Results

### Characterization of human ECO.

**Figure 1:**
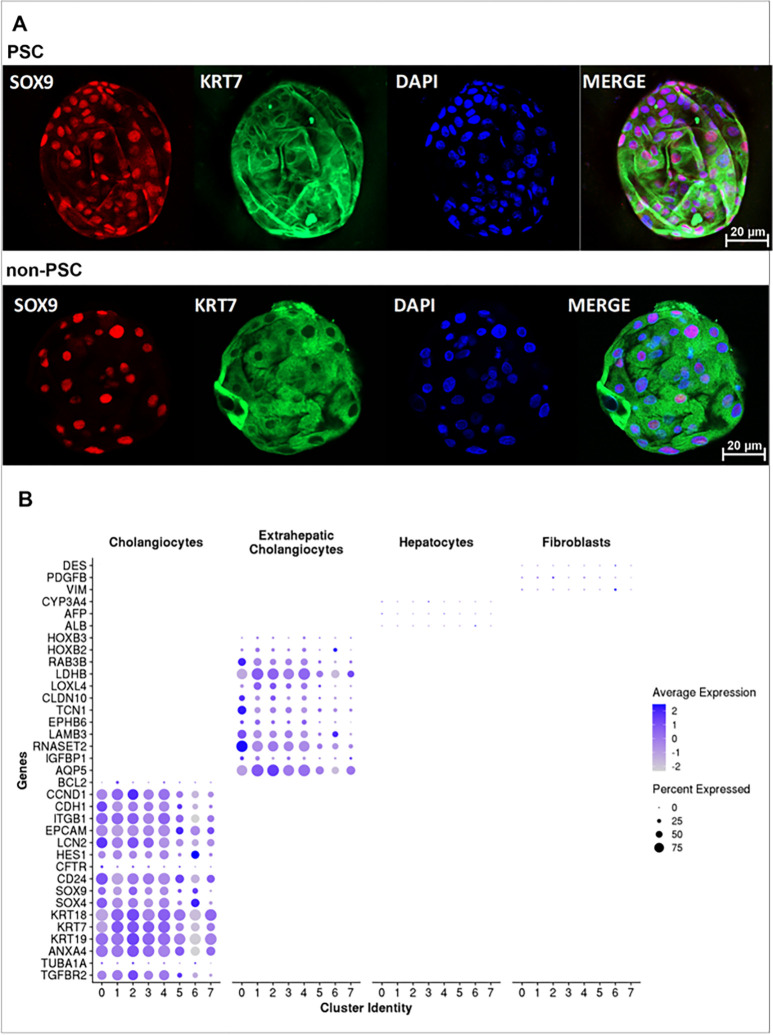
Characterization of patient-derived ECO. **A)**Whole mount immunofluorescence of PSC and non-PSC ECO stained with SOX9 (red), KRT7 (green), and DAPI (blue) obtained by confocal (objective =20x, scale bar=20 μm). **B)** Dot plot displaying expression of known classical cholangiocyte, extrahepatic bile duct, hepatocyte, and fibroblast marker genes in each cholangiocyte cluster identified by single cell RNA seq analysis of ECO. The intensity of the color indicates the average expression, and the size of the circle is directly proportional to the percentage of cells expressing each gene.

We obtained ECO from PSC (n = 9) and non-PSC (n = 7) patients. Patient characteristics and indications for the ERC are described in Table 1. To confirm the cholangiocyte phenotype of the ECO, we initially performed whole mount immunofluorescence for SRY-box transcription factor 9 (SOX9) and cytokeratin 7 (KRT7), both expressed uniquely in cholangiocytes within the liver. SOX 9 and KRT7 were expressed in both PSC and non-PSC patient derived ECO and appeared to be expressed by all cells within the organoids (Fig. 1A). Next, we performed scRNA-seq of the PSC (n = 4) and non-PSC (n = 4) patient-derived ECO. Cholangiocyte gene expression profiles were strongly enriched in all clusters [cytokeratin 7 (KRT7), 18 (KRT18) and 19 (KRT19); Epithelial cellular adhesion molecule (EPCAM)], whereas genes expressed in hepatocytes and/or fibroblasts, but not cholangiocytes were not significantly expressed in any of the clusters [albumin (ALB), alpha-fetoprotein (AFP), cytochrome P450 family 3 subfamily A member 4 (CYP3A4), desmin (DES), platelet-derived growth factor subunit B (PDGFB), and vimentin (VIM)]. Genes previously identified in ECO and extrahepatic bile ducts were also expressed by all clusters [homeobox B2 (HOXB2), homeobox B3 (HOXB3), aquaporin 5 (AQP5), insulin like growth factor binding protein 1 (IGFBP1), ribonuclease T2 (RNASET2), laminin subunit beta 3 (LAMB3) and lactate dehydrogenase B (LDHB)] (Fig. 1B). ^[2, 9, 10]^ These observations verify the extrahepatic cholangiocyte phenotype of the cells within the ECO and are consistent with the observations of others. ^[9, 10]^

### Characterization of cholangiocyte genetic heterogeneity by scRNA-seq.

**Figure 2:**
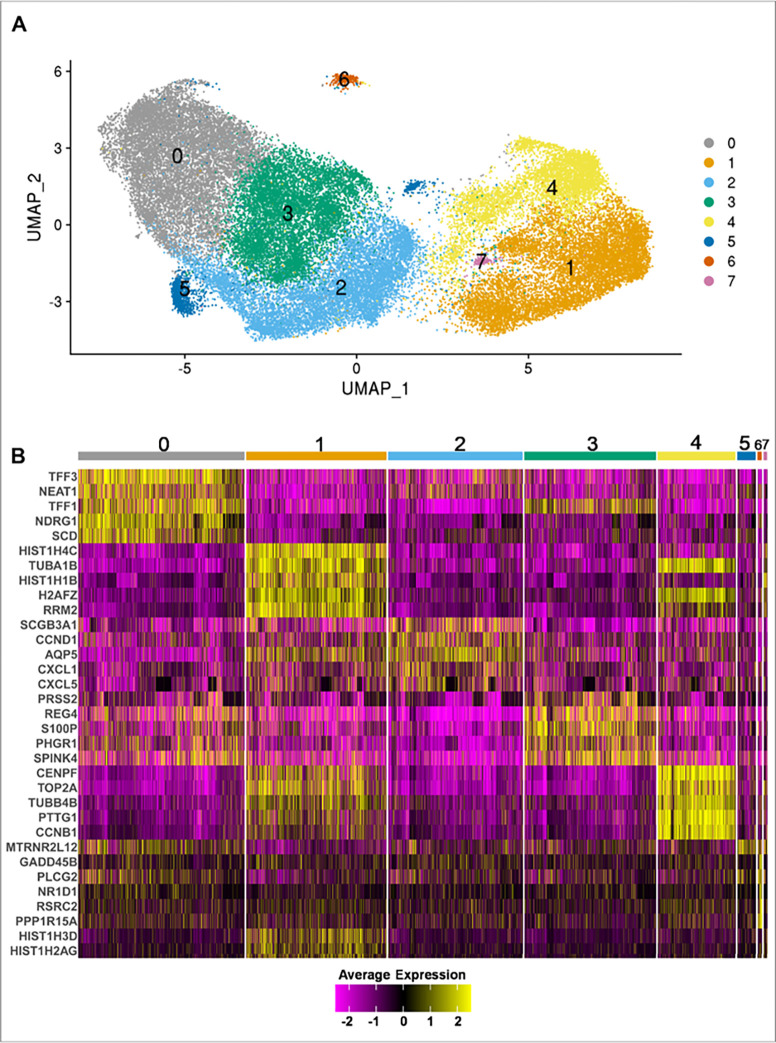
Identification of distinct cholangiocyte clusters in ECO. ECO from PSC (n=4) and non-PSC (n=4) patients were subjected to single cell RNA seq. **A)** UMAP plot visualizing eight distinct clusters termed Cluster (0) to (8), obtained by unsupervised clustering. Colors represent a unique cell population cluster as identified by transcriptional signature. **B)** Heatmap displaying the top 5 cluster marker genes for each unique cell population cluster. Colors indicate the expression of each gene in a cluster, when compared to all other cluster combined (Yellow = upregulation, Purple = downregulation).

Given the known genetic heterogeneity of cholangiocytes, we examined genetic markers in the PSC and non-PSC ECO. Interrogating scRNA-seq data, we identified eight clusters shared between the ECO derived from all samples. (Fig. 2A, Fig. S1). The top 5 conserved cluster marker genes are displayed as a heatmap in Fig. 2B. Cluster 0 was characterized by an enhanced expression of genes associated with mucosal maintenance (TFF31, TFF2, TFF3), hypoxia (NDRG1, EGLN3, CA9), reactive oxygen species (ERO1A, DUOX2, and its maturation factor DUOXA2) and two long non-coding RNA genes (MALAT1, NEAT1) (Fig. 2B, Suppl.File.1). Clusters 1 and 4 had increased expression of MKI67 and PCNA suggesting an active proliferative state in these clusters at the time of the analysis (Suppl.File.1). Interestingly, Cluster 2 expressed TNFSF15, TNFRSF12A, TNFAIP2, CXCL1, CXCL5, CXCL8, CCND1, and IL-18, consistent with an inflammatory phenotype^[32]^ (Fig. 2B, Suppl.File.1). Cluster 5 and Cluster 7 had a limited number of conserved cluster marker genes. Cluster 5 had only one conserved cluster marker gene, MT-RNR2 Like 12 (MTRNR2L12), which is a mitochondrial-derived peptide that exerts anti-apoptotic effects by preventing the translocation of Bax from cytosol to mitochondria^[33]^. Cluster 7 had three conserved markers, all of them belonging to histone coding genes (HIST1H1B, HIST1H3D, HIST1H2AG) (Fig. 2.B). Lastly, Cluster 6 had increased expression of genes associated with cell damage such as GADD45B, GADD45G, PPP1R15A, and HSPB1 (Fig. 2B, Suppl.File.1), which likely indicates that the cells in this cluster are manifesting a stress response. Altogether, the identification of different cell clusters confirms the heterogeneity of extrahepatic cholangiocytes.

### Transcriptomic profiling of non-PSC and PSC ECO demonstrate differences between the two groups.

**Figure 3:**
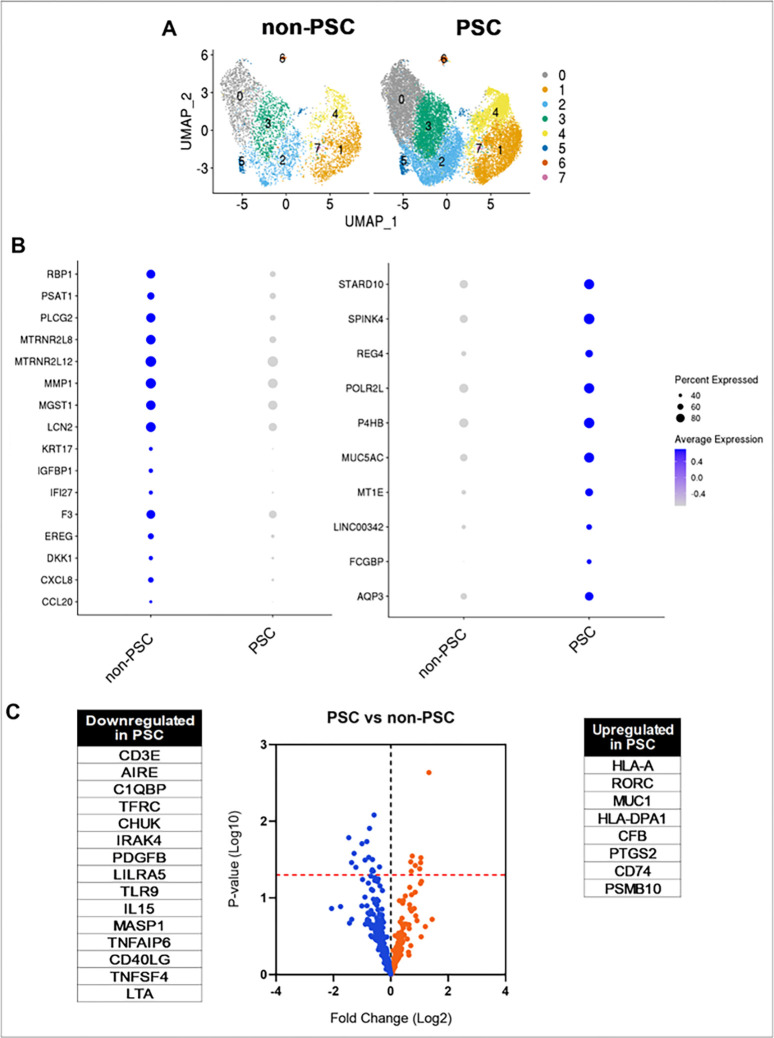
Transcriptomic profiling of PSC and non-PSC ECO. **A)** UMAP plots comparing clusters from PSC (n=4) and non-PSC (n=4) ECO. **B)** Dot plots visualizing DEG between PSC and non-PSC ECO. Color indicates average expression; size of the circle indicates percentage of cells expressing each gene. DEG were determined by a p-value <0.05, Log2Fold change >0.2, and their presence in at least four clusters. **C)** Volcano plot comparing PSC (n=6) and non-PSC (n=5) ECO by NanoString analysis. Dots represent a downregulated (blue) or upregulated (orange) gene. Line (red) indicates p = 0.05. Statistically significant genes are listed next to volcano plot.

Once the clusters were identified and characterized, we elected to investigate whether a cluster or multiple clusters were different between PSC and non-PSC ECO by comparing the cell percentage of each cluster. However, no significant differences were identified (Fig. 3A and Fig. S2). These data suggests that PSC ECO do not have a unique and characteristic cell population of cholangiocytes when compared to non-PSC ECO, but rather share the same cholangiocyte populations. Nonetheless, there can be differences in expression of genes shared amongst clusters that do not distinguish individual clusters per se, but yet differ between PSC and non-PSC ECO. Therefore, we analyzed the transcriptional profiles of the groups by examining DEG between PSC and non-PSC ECO. DEG analysis led to the identification of genes that were consistently enriched in PSC ECO and in non-PSC ECO in the majority of the clusters. The main enriched genes in PSC ECO were found to be AQP3, FCGBP, LINC00342, MT1E, MUC5AC, P4HB, POLR2L, PPIB, REG4, SPINK4, and STARD10. (Fig. 3B). On the other hand, non-PSC ECO demonstrated enrichment of CCL20, CXCL8, DKK1, EREG, F3, IFI27, IGFBP1, KRT17, LCN2, MGST1, MMP1, MTRNR2L12, MTRNR2L8, PLCG2, PSAT1, and RBP1 (Fig. 3B) This data confirms that differences in gene expression exist throughout cholangiocyte populations when comparing PSC and non-PSC ECO.

We additionally elected to investigate the differences in expression of inflammation-related genes between PSC and non-PSC ECO by NanoString analysis given that different methodologies yield complementary results. These data demonstrated that PSC ECO have a higher expression of HLA-A, RORC, MUC1, HLA-DPA1, CFB, PTGS2, CD74 and PSMB10 when compared to non-PSC (Fig. 3C). Downregulated genes in PSC ECO included CD3E, AIRE, C1QBP, TFRC and CHUK (Fig. 3C). These results indicate a baseline difference in gene expression between PSC and non-PSC ECO and their respective cholangiocyte populations. Interestingly, both PSC and non-PSC ECO express inflammation associated genes, but the specific genes differ between the two groups.

### Secretome analysis reveals increased secretion of pro-inflammatory proteins by PCS ECO.

To further understand the differences between PSC and non-PSC ECO at baseline, we investigated secreted inflammation-related proteins by performing Olink analysis on the supernatant of the ECO. Although both PSC and non-PSC ECO secreted inflammatory proteins, PSC ECO had a significantly higher release of proteins that included cytokines and chemokines such as IL-6, TRAIL, CXCL9, IL-2, CCL4, MCP-4, TNFSF14, IL-13, MCP-2, CCL25, and IL-5 (Fig. S3). These data indicates that PSC and non-PSC ECO both secrete inflammatory proteins, however the secreted protein abundance for most of these proteins was greater in PSC ECO.

### PSC and non-PSC patient derived ECO respond differently to IL-17A stimulation.

**Figure 4:**
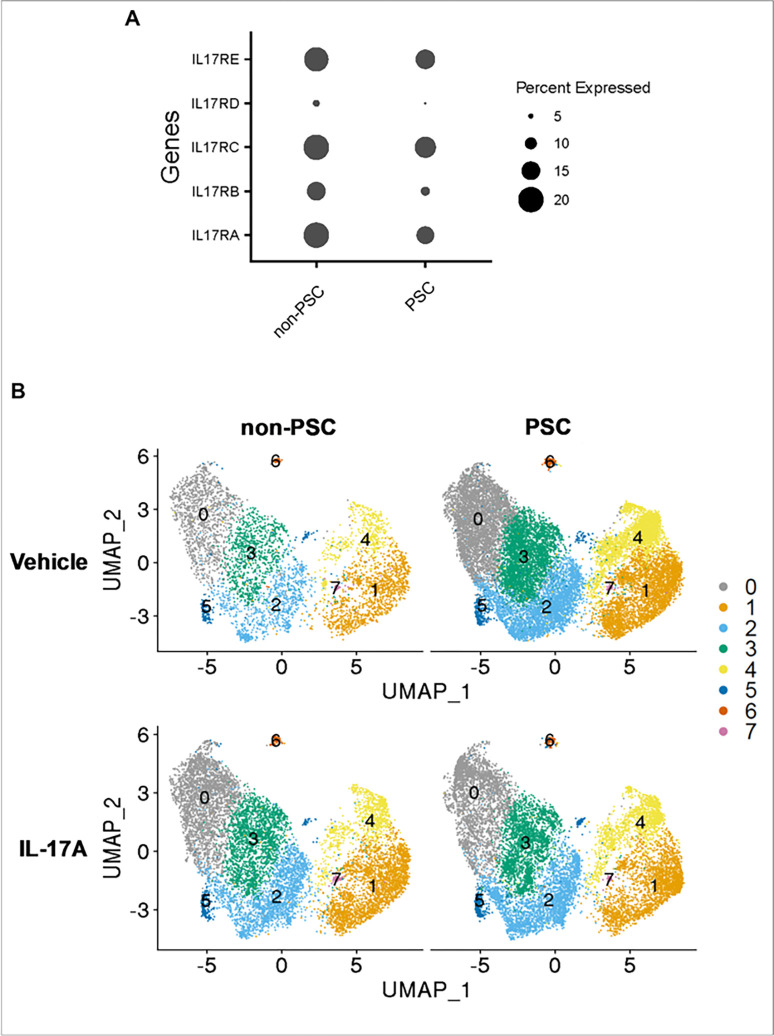
IL-17 response in PSC and non-PSC ECO. PSC and non-PSC ECO were treated with vehicle or IL-17A (100ng/ml) for 24 hr. and subjected to single cell RNA seq. **A)** Dot plot displaying IL-17 receptor family expression represented as a pseudo bulk analysis. The size of the circle indicates the percentage of cells expressing each gene. **B)** UMAP plots visualizing the different clusters in PSC and non-PSC, and treatments (Vehicle and IL-17A). Colors represent unique cell population clusters as identified by transcriptional signature.

The initial step during the IL-17 signaling pathway requires the binding of the IL-17 ligand family to its cognate receptors.^14^ Therefore, to further characterize the ECO and ensure that cholangiocytes expressed the requisite receptor(s) for ligand binding, we analyzed such expression on scRNA-seq data as a pseudo bulk analysis. Both non-PSC and PSC ECO demonstrated a similar expression of the receptor family, with ILRA, ILRC and IL17RE being more abundantly expressed. Hence, both PSC and non-PSC patient-derived ECO express the requisite cognate receptor subunits to initiate IL-17A signaling (Fig. 4A).

To define the direct effects of IL-17A treatment in PSC and non-PSC ECO, the ECO were stimulated with IL-17A, and scRNA-seq was performed. Initially, the cell percentage of each cluster in PSC and non-PSC ECO after the treatment was evaluated. However, no significant differences were identified within samples (Fig. 4B, Fig. S2). Hence, IL-17 treatment does not induce changes in cell ratios in different cholangiocyte cluster populations when comparing non-PSC and PSC ECO.

**Figure 5:**
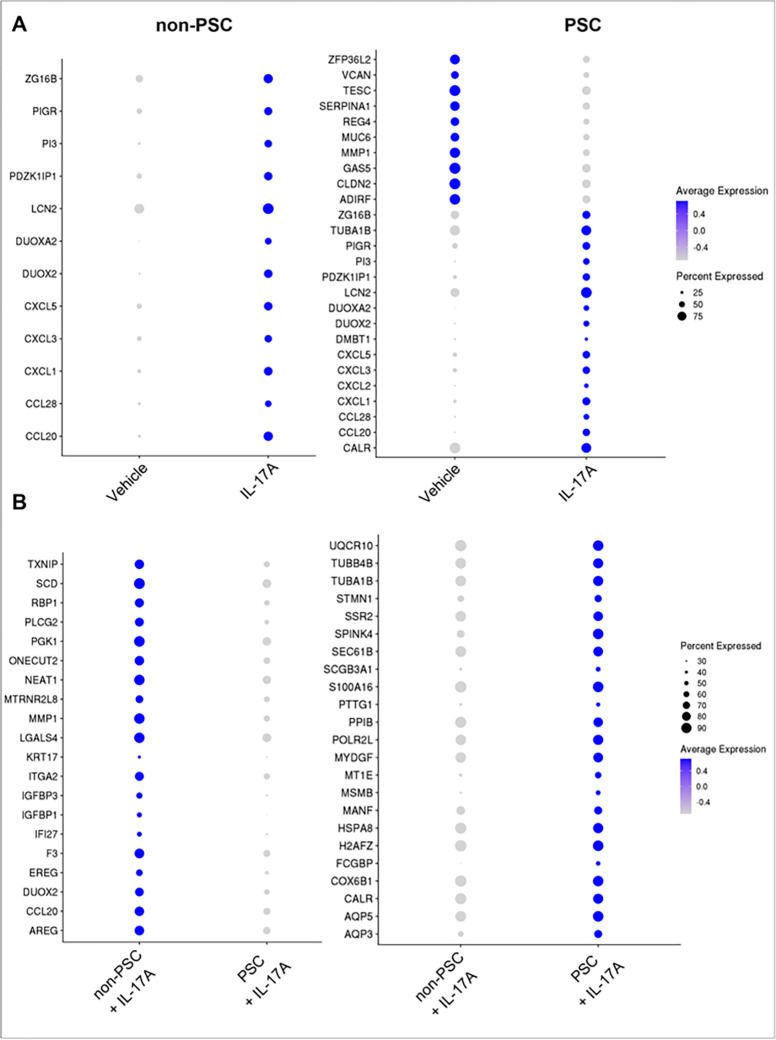
Differential response to IL-17A stimulation between non-PSC and PSC ECO by scRNA-seq. **A)** Dot plots displaying pseudo bulk DEG between ECO treated with vehicle and IL-17A within each condition (PSC vs non-PSC). **B)** Dot plots displaying DEG between PSC and non-PSC ECO treated with IL-17A. The intensity of the color indicates the average expression, and the size of the circle indicates the percentage of cells expressing each gene. DEG were determined by a p-value<0.05 and Log2Fold change > 0.2, and their presence in at least four clusters.

Next, the DEG between vehicle and IL-17A-treated ECO within each group (PSC ECO ± IL-17A, non-PSC ECO ± IL-17A) were investigated, enabling the identification of genes that are either upregulated or downregulated by treatment in ECO. Both PSC and non-PSC ECO shared a common response to IL-17A, displaying changes in expression of CCL20, CCL28, CXCL1, CXCL3, CXCL5, DUOX2, DUOXA2, LCN2, PDZK1IP1, PI3, PIGR, ZG16B (Fig. 5A). However, there were also differences in genetic expression between the two cohorts. In particular, PSC ECO had an increased number of DEG after the treatment with IL-17A, and a significant number of these genes did not display expression changes in the non-PSC ECO (Fig. 5A). These results imply that genetic regulation by IL-17A is different between non-PSC and PSC ECO.

To further understand the differences between PSC and non-PSC ECO, IL-17A treated ECO only (IL-17A treated PSC vs IL-17A treated non-PSC ECO) were selected and DEG were investigated between these two cohorts. In this instance, the analysis enabled the identification of genes that are significantly different between PSC and non-PSC after treatment with IL-17A. PSC ECO displayed enrichment of AQP5, CALR, COX6B1, H2AFZ, HSPA8, MANF, MSMB, MYDGF, PTTG1, S100A16, SCGB3A1, SEC61B, SSR2, STMN1, TUBA1B, TUBB4B, and UQCR10. (Fig. 5B). Similarly, non-PSC ECO displayed enrichment of AREG, DUOX2, IGFBP3, ITGA2, LGALS4, NEAT1, ONECUT2, PGK1, SCD, and TXMP (Fig. 5B). These results suggest that PSC ECO respond differently to IL-17A stimulation compared to non-PSC ECO.

**Figure 6:**
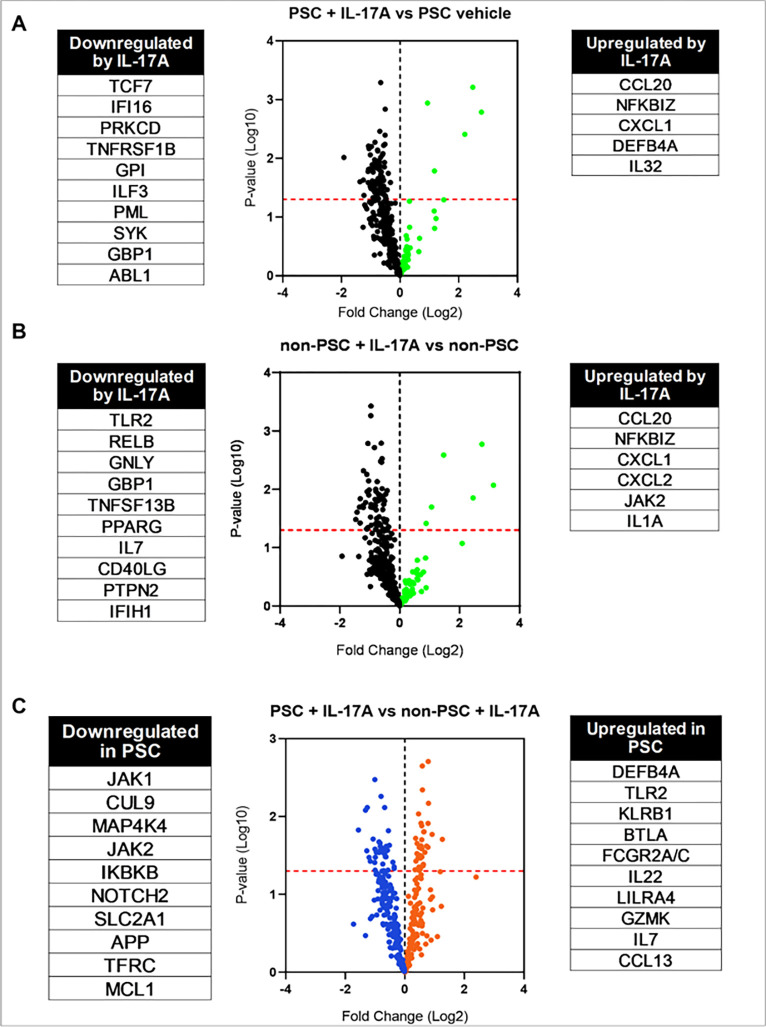
IL-17A response throughout distinct cholangiocyte cluster populations in non-PSC and PSC ECO. Volcano plots displaying mRNA expression profiles obtained by NanoString analysis comparing **A)** vehicle- vs. IL-17A-treated PSC ECO, **B)** vehicle- vs. IL-17A-treated non-PSC ECO (upregulated genes in green, downregulated genes in black), **C)** IL-17A-treated PSC vs. non-PSC ECO (upregulated genes in orange, downregulated genes in blue). Each dot represents a gene. Upregulated and downregulated genes are listed next to the volcano plots.

In addition to scRNA-seq, we again performed NanoString analysis and investigated inflammation-related genes after treatment with IL-17A. Initially, we investigated the effects of the treatment by comparing vehicle and IL-17 treated ECO. This confirmed the upregulation of CCL20 and CXCL1 (Fig. 6A, B) in both ECO. In particular, PSC ECO displayed upregulation of DEFB4A and IL-32, which was not present in non-PSC ECO, making the upregulation of these genes unique to PSC (Fig. 6A). Similarly, non-PSC ECO displayed upregulation of JAK2 and IL-1A after treatment with IL-17A (Fig. 6B). Interestingly, IL-17 treatment appears to downregulate more than 30 genes in both ECO. When comparing IL-17 treated cells, PSC ECO expressed upregulation of inflammation-related genes such as DEFB4A, TLR2, KLRB1, BTLA, FCGR2A/C and IL22, LILRA4, GZMK, IL7 and CCL13 (Fig. 6C). In a similar manner, PSC ECO expressed downregulation of JAK1, CUL9, MAP4K4, JAK2 and IKBKB (Fig. 6C.). Lastly, we performed Olink analysis on the supernatant of both PSC and non-PSC ECO to identify changes in secreted proteins. When comparing treated cells only, PSC ECO appear to have a higher secretion of various cytokines and chemokines such as MCP-3, IL7, CXCL11, CXCL9, CCL11, IL-10, TNF, CXCL6, IFN-gamma, CCL25, TWEAK, IL5, and TNFB (Fig. S4). Taken together, these results imply that the response to IL-17A is different between PSC and non-PSC ECO at the RNA and protein level, suggesting a role for this signaling pathway in PSC pathogenesis.

### Somatic variants in PSC ECO.

**Figure 7:**
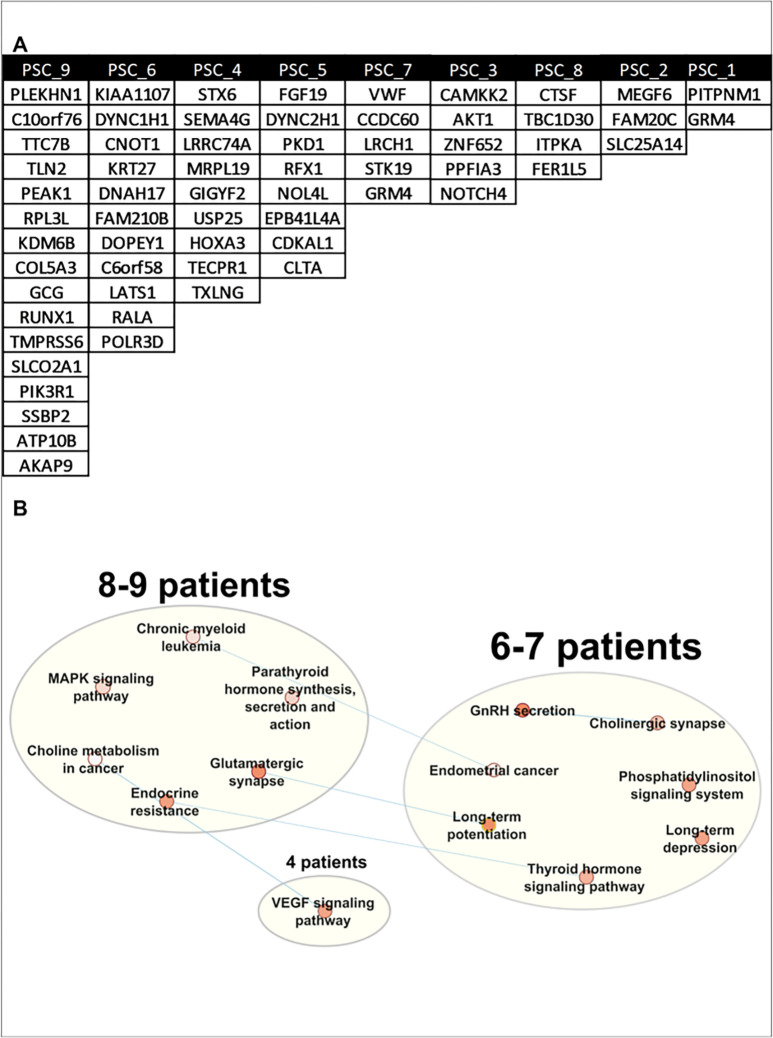
Identification of rare somatic variants in PSC ECO. WGS was performed in nine PSC patient-derived ECO. A) Table displaying somatic variants with CADD>25 by PSC ECO. **B)** Somatic variant enriched pathways network of PSC ECO. Relaxed variant pathway analysis via Cytoscape and AutoAnnotate. Patients with variants in similar pathways are grouped. Each pathway is represented by a dot with a color indicating significance: white (p < 0.05) to red (p < 0.0001). Blue lines indicate pathways that share genes that contain a somatic variant.

To investigate whether the differences in response to IL-17A between the ECO could be linked to a specific mutational signature, we examined somatic mutations based on the previously published work on ulcerative colitis and the IL-17 signaling pathway in the PSC ECO.^31^ Somatic variants were found by excluding confirmed germline variants in ES from peripheral white blood cells. Potentially deleterious variants, with a CADD score^[34]^ higher than 25, were identified in all the patients, with a number of variants ranging from 2 to 16 (Fig. 7A). However, none of the rare variants were in genes directly associated with the IL-17A signaling pathway. Acknowledging the fact that filtering in only variants with a high CADD score excludes somatic variants that might have an impact in the protein, we relaxed the filtering criteria (Fig.S5) and performed KEGG pathway analysis^[35]^. Each of the patients had one or more rare somatic variants within at least one gene in several enriched pathways (Fig. 7B). However, variants within the MAPK signaling pathway were driving the enrichment of these pathways and are depicted in Fig. S6 and S7. Taken together, these data confirm the presence of somatic variants in PSC organoids. However, none of the rare variants were in genes related to the IL-17 signaling pathway. Of note, a limitation of this analysis is in comparing GS in the ECO to WES in PWBC.

## Discussion

The results of this study provide insight into differential signaling of IL-17A in ECO derived from PSC patients. The major and significant findings of this study are as follows: i) both non-PSC and PSC ECO demonstrated similar cholangiocyte heterogeneity; ii) ECO derived from PSC and non-PSC patients both express IL-17 receptors A, C, and E; iii) ECO from PSC and non-PSC patients respond differentially to IL-17 stimulation with different gene expression and secretome; iv) no rare somatic variants were identified in genes associated with the IL-17A signaling pathway in PSC ECO, although rare somatic variants regulating the MAPK signaling pathway were identified. Taken together the data suggest IL-17A signals differently in PSC vs. non-PSC patient derived cholangiocytes. These results are described in further detail below.

To get further insight into the PSC and non-PSC ECO, in an unbiased manner, and to characterize them in-depth, we applied scRNA-seq technology. This allowed us to examine cholangiocyte heterogeneity and led to the identification of 8 different clusters. However, the clusters were not different between PSC and non-PSC ECO, and neither did the cluster number or cell percentage of each cluster change with IL-17A stimulation. In another human cholangiopathy termed primary biliary cholangitis (PBC) a select population of cholangiocytes identified by the expression of DUOX and ACE2 were noted to be absent in PBC as compared to controls^[36]^. Comparable changes in PSC do not appear to occur as assessed by scRNA-seq of ECO. Indeed, we identified a DUOX2 positive population in ECO (data not shown); However, the expression of ACE2 was not observed in either PSC or non-PSC ECO derived from the large bile duct; and ACE2 expression appears to be limited to the intrahepatic cholangiocytes. This observation may reflect differences in the pathogenesis of the two diseases where PBC is an autoimmune mediated ductopenic disease of intrahepatic cholangiocytes whereas PSC an inflammatory fibro-obliterative disease of extrahepatic and intrahepatic cholangiocytes.

It has previously been demonstrated that cholangiocytes respond to IL-17 stimulation, suggesting the presence of this cytokine’s cognate receptors throughout the biliary tree.^[29]^ However, the presence of and differences in expression of these receptors had yet to be characterized in human cholangiocytes. In this study we examined the expression of the IL-17 receptor subunits A-E in PSC and non-PSC patient derived ECO. Our data suggest that IL-17RA, IL-17RC and IL-17RE were equally expressed in both PSC and non-PSC ECO. Hence, differences in IL-17 receptor expression are unlikely to account for differences in IL-17 signaling between PSC and non-PSC ECO, or in PSC disease pathogenesis.

Once we confirmed that cholangiocytes express IL-17A receptors, we treated our PSC and non-PSC ECO with IL-17A and investigated differences in gene expression. Both PSC and non-PSC ECO responded to IL-17A with gene expression indicating IL-17A signaling was intact. Differences in response to IL-17A stimulation between PSC and non-PSC ECO were identified by different complementary techniques including scRNA-seq, NanoString analysis, and quantification of the secretome which confirmed that the IL-17A signaling pathway is perturbed in PSC ECO. However, unlike IBD^[31]^, we did not identify rare somatic variants directly involving genes that are known to regulate the IL-17 signaling pathway. Nonetheless, rare somatic variants potentially regulating the MAPK signaling pathway were identified. Interestingly the activation of the MAPK signaling pathway has been previously demonstrated to be a downstream effect of IL-17A stimulation^[37]^; however, how these somatic gene alterations modulate specific IL-17 stimulation in cholangiocytes remains to be explored. We also speculate that there might be epigenetic changes in genes regulating the IL-17 signaling pathway, environmental factors or effects caused by a combination of multiple polymorphisms driving this different response. Studies of the epigenome and the functional studies of the rare somatic variants potentially regulating the MAPK pathway are beyond the scope of this study but should be further examined.

In conclusion, we examined the response of PSC vs non-PSC ECO to IL-17A stimulation. Although scRNA seq-based cluster analysis did not identify unique PSC clusters, IL-17A simulation uncovered differential gene expression and alterations in the secretome between the two patient populations. These observations are consistent with the concept that alteration of IL-17 signaling may contribute to the pathogenesis of PSC. These results also highlight the utility of ECO in examining disease mechanisms in PSC and suggest IL-17 directed therapy should be further explored in PSC.

## Materials And Methods

### Materials.

Advanced Dulbecco’s modified Eagle’s medium/F12, Antibiotic-Antimycotic (Anti-Anti), and B-27 supplement were from Thermo Fisher Scientific (Waltham, MA); recombinant human R-spondin-1, recombinant human EGF, recombinant human HGF, recombinant human FGF10, and recombinant human noggin were from PeproTech (Cranbury, NJ):, gastrin, N-acetylcysteine, Y27632, nicotinamide and A83-01 were from Millipore-Sigma (Burlington, MA); human recombinant Wnt3A, Forskolin and human recombinant IL-17A were from R&D Systems (Minneapolis, MN). Matrigel (354230) and cell recovery solution were from Corning (Kennebunk, ME, USA).

### Patient enrollment and tissue collection.

Patients with PSC were diagnosed using criteria established by the American Association for the Study of Liver Disease (AASLD) guidelines^[38]^. Patients, with PSC or non-PSC, who were undergoing clinically indicated endoscopic retrograde cholangiography (ERC), were identified through the electronic medical record. (Table 1). Patients with known malignancy, orthotopic liver transplantation, a history of biliary-enteric anastomosis, or other chronic liver diseases, were excluded from this study. The study, including sample collection, was approved by the Mayo Clinic Institutional Review Board, all research was performed in accordance with relevant guidelines/regulations and informed consent was obtained from all subjects and/or their legal guardian(s) prior to ERC.

Following specific cannulation of the common bile duct, bile was aspirated via a catheter. Up to 10 ml of bile and/or the brush following cytology of extrahepatic bile ducts were collected and placed on ice prior to processing. Once processed, the samples were used for extrahepatic cholangiocyte organoid (ECO) generation within the first hour after being collected.

### Organoid generation and culture.

Bile samples were diluted 1:10 with ice-cold PBS + Anti-Anti (1:100 dilution) and processed as previously described by Soroka et al.^2^ The cytology brush was placed in a sterile polystyrene Petri dish containing PBS + Anti-Anti (1:100 dilution) and a sterile pipette was used to mechanically remove the cellular material embedded in the brush. The sample was transferred to a 15 ml conical tube and centrifuged at 300 g for 5 min, then the supernatant was removed, and the resulting pellet was washed twice with ice cold PBS + Anti-Anti and once with advanced Dulbecco’s modified Eagle’s medium/F12. The cell pellets from both brushing and bile samples were resuspended in Matrigel, dispensed onto 48-well plates as 30-μl droplets and grown in organoid complete medium.^2^ After three days, organoid expansion medium defined by Soroka et al.^2^ was employed to expand the organoids and was refreshed every two days; ECO were passaged when approx. 75% confluent with a split ratio of 1:3/1:4 using Cell Recovery Solution on ice for 30 minutes.

### Organoid immunofluorescence.

ECO were grown in 30-μl matrigel domes in 35 mm glass bottom Petri dishes (GBD00004-200; Cell E&G LLC, San Diego, CA). Whole mount immunofluorescence for cytokeratin 7 (KRT7) and SRY-Box transcription factor 9 (SOX9), both cholangiocyte markers, was performed as previously described.^[39]^ Mouse monoclonal anti-KRT7 antibody (#sc-400628, Santa Cruz Biotechnology, Santa Cruz, CA) and rabbit monoclonal anti-SOX9 antibody (D8G8H, #82630, Cell Signaling, Danvers, MA) were diluted 1:100. Secondary antibodies, goat anti-mouse IgG AlexaFluor488 and chicken anti-rabbit IgG AlexaFluor594 (Invitrogen/Thermo Fisher Scientific) were used for KRT7 and SOX9, respectively, at a 1:200 dilution. 4′,6-diamidino-2-phenylindole (DAPI, 300 nM) was added together with the secondary antibodies to visualize the nuclei. The slides were mounted with ProLong^™^ Gold Antifade Mountant (Thermo Fisher Scientific) and analyzed by confocal microscopy using ZEISS LSM 980 (Zeiss, Oberkochen, Germany).

### NanoString analysis.

ECO from PSC (n = 6) and non-PSC patients (n = 5) were treated with vehicle or recombinant human IL-17A (100 ng/mL) for 24 hours. Total RNA was extracted using RNeasy^®^ Plus mini kit (Qiagen, Hilden, Germany), and 100 ng of RNA was analyzed to determine the expression of a panel of immune-related genes using the nCounter_Human Immunology V2 Panel (NanoString Technologies, Seattle, USA) according to the manufacturer’s instructions, employing an nCounter MAX analysis system.

### Single-cell RNA-seq analysis.

ECO from PSC (n = 4) and non-PSC patients (n = 4), all at passage 3, were treated with vehicle or human recombinant IL-17A (100 ng/mL) for 24 hours. The organoids underwent trypsin digestion followed by mechanical dissociation to obtain single cell suspensions in PBS + 0.1% BSA and were submitted to the Gene Analysis Core of the Medical Genome Facility (Mayo Clinic, Rochester, MN) for scRNA-seq analysis. Cell number and viability were measured using the Vi-Cell XR Cell Viability Analyzer (Beckman-Coulter, Brea, CA). The cDNA master mix was prepared according to the manufacture’s instruction for Chromium Next GEM Single Cell 3’ Library and Gel Bead Kit (10x Genomics, Pleasanton, CA). The standard targeted cell recovery was set to ~ 3000 cells. All cDNA pools and resulting libraries were measured using Qubit High Sensitivity assays (Thermo Fisher Scientific, Waltham, MA) and Agilent Bioanalyzer High Sensitivity chips (Agilent, Santa Clara, CA). Libraries were sequenced at between 40,000 and 50,000 fragment reads per cell following Illumina’s standard protocol using the Illumina cBot and HiSeq 3000/4000 PE Cluster Kit (Illumina, San Diego, CA). The flow cells were sequenced as 100 X 2 paired end reads on an Illumina HiSeq 4000 HD using HiSeq 3000/4000 sequencing kit and HCS v3.4.0.38 collection software. Base-calling was performed using Illumina’s RTA version 2.7.7.

After sequencing, the ECO samples were processed using 10x Genomics Cell Ranger 5.0.0 ^[40]^. Each sample’s aligned reads were then imported into R for quality control processing by Seurat^[41]^ (v4), filtering cells with less than 200 nCounts or greater than 40% mitochondrial reads. All samples were then normalized by SCTransform (v2) and integrated using Harmony (v0.1.1)^[42]^. Analyses were performed following Seurat recommendations. Filtering of marker differentially expressed genes was performed using a q-value < 0.05, an |log_2−_fold change| > 0.2, and a custom parameter: percent expression change ratio > 0.2 (when comparing treatments or conditions). The percent expression change ratio was calculated using the percent of cells showing expression of the feature in the groups being compared (pct.1/pct.2 or pct.2/pct1 with the greater being the numerator). Code for all analyses is available upon request.

### Genome sequencing (GS).

ECO from 9 PSC patients were subjected to GS. DNA isolation was performed using the QIAamp Blood Mini Kit (Qiagen). Samples were submitted to the Gene Analysis Core of the Medical Genome Facility (Mayo Clinic, Rochester, MN) for GS. Libraries were prepared using up to 500 ng genomic DNA according to the manufacturer’s instructions for the Nextera DNA Flex Library Prep Kit (Illumina, San Diego, CA). The concentration and size distribution of the completed libraries were determined using the Fragment Analyzer (Agilent, Santa Clara, CA) and Qubit fluorometry (Invitrogen, Carlsbad, CA). Libraries were sequenced at an average coverage of approximately 40x following Illumina’s standard protocol for the Illumina NovaSeq 6000 and S2 flow cell. The flow cells were sequenced as 150 × 2 paired end reads using the NovaSeq S2 v1.5 sequencing kit and NovaSeq Control Software v1.7.0. Base-calling was performed using Illumina’s RTA version 3.4.4.

To differentiate between somatic and germline variants, the results of the GS were compared to the data obtained from previous exome sequencing (ES) on the patient’s peripheral white blood cell (PWBC) samples. Isolation of genomic DNA from blood samples was performed by the Biospecimens Accessioning and Processing laboratory at the Mayo Clinic using the PureGene kit (Gentra Systems, Minneapolis, MN, USA) as specimens were received and submitted to the Gene Analysis Core of the Medical Genome Facility (Mayo Clinic, Rochester, MN) for ES. Paired-end libraries were prepared with approximately 400 ng of genomic DNA using the SureSelect XT Low Input Reagent Kit (Agilent, Santa Clara, CA). The concentration and size distribution of the completed libraries were determined using an Agilent Bioanalyzer DNA 1000 chip or Advance Fragment Analyzer and Qubit fluorometry (Invitrogen, Carlsbad, CA). Adaptor-ligated DNA was amplified with the SureSelect Post-Capture forward and specific index reverse primers for 12 cycles. Exon capture was carried out using 750 ng of the prepared library following the protocol for Agilent’s SureSelect Human All Exon v5 + UTRs 75 MB kit. The concentration and size distribution of the completed captured libraries were determined on Qubit (Invitrogen) and an Agilent Bioanalyzer DNA 1000 chip. Libraries were sequenced, yielding approximately 43 million to 59 million read pairs per sample, following Illumina’s standard protocol for the Illumina NextSeq 2000. The NextSeq P2 flow cell was sequenced as 150 × 2 paired end reads using the NextSeq 1000/2000 Control Software v1.4.1 and RTA3.

### Somatic variant analysis.

Somatic variants were called using both Mutect2^[43]^ and Strelka^[44]^ variant callers. The reference samples were exome sequenced bam files and the somatic were genome sequenced bam files, both aligned to the hg38 human genome. Default parameters were used for both tools. Somatic variant calls were filtered on the ‘PASS’ flag from Mutect2 and Strelka variant callers, a read depth of 10 reads in both the GS tumor and ES normal/reference sample. Somatic variants were further annotated using BioR^[45]^ variant annotation with information from CAVA^[46]^ and 1000 genomes^[47]^, ExAC^[48]^, and gnomAD^[49]^ databases. Variants with a population allele frequency of less than 0.0001 were kept as rare variants. Any variant with a ‘likely artifact’ designation in gnomAD was removed. Finally, variants were annotated with a CADD score^[34]^ to quantitate the predicted impact of the variant, keeping variants with a CADD score greater than 25 (Fig.S5A.). CADD was chosen over other prediction tools because it integrates multiple annotations into one metric and includes splicing scores and annotations related to non-coding regions of the genome. Relaxed variants were obtained by excluding impact factor and CADD score from filtering criteria (Fig.S5B) Relaxed variants were input into Cytoscape^[50]^ for pathway enrichment and visualization using Enrichment Map and AutoAnnotate. KEGG and GO pathways with a p-value < 0.05 were selected for plotting.

### Secretome analysis by Olink analysis.

ECO from PSC (n = 5) and non-PSC (n = 7) patients at passage 4–6 were treated with IL-17A (100 ng/ml) or vehicle in expansion medium. After 24 hr., medium was removed, centrifuged at 1,500 × g for 5 min and the supernatants stored at −80°C until used. In addition, organoid expansion medium exposed to matrigel without organoids for 24 hr. was used as negative control. The organoids were collected, centrifuged at 300 × g for 5 min, and lysed in RIPA buffer (50 mM TRIS-HCl, pH 7.4, 1% NP-40, 0.25% NaDCA, 1 mM EDTA, 1 mM Na_3_VO_4_, 1 mM NaF, protease inhibitor mix). Protein content was measured by BCA assay (Thermo Fisher) and used as normalization factor. ECO supernatants were plated in a randomized fashion and shipped to the University of Minnesota Genomic Center (Oakdale, MN) where they were analyzed by proximity extension technology (Olink, Uppsala, Sweden) using the Olink^®^ Target 96 Inflammation panel. Data were expressed as normalized protein expression (NPX) values. To account for the variability in organoid number across samples, and the proteins contained in the medium and matrigel, adjusted NPX values were calculated by subtracting the corresponding NPX value from the negative control (baseline) for each protein and normalizing to the −Log2 of the organoid protein content.

## Figures and Tables

**Table 1:** Baseline characteristics of the patients included in the study at the time of ERC.

Patient	Diagnosis	Gender	Age	IBD	IBD Type	Cirrhosis	Indication for ERC	Anatomical location of sample	T Bili (mg/dL)	Alk Phos (92-279 IU/L)
1	PSC, AIH	F	68	No	-	Yes	Stent removal	Common hepatic duct, right main duct	3.7	583
2	PSC	F	48	Yes	UC	Yes	Elevated bilurribine	Distal Common Bile Duct	2.6	37
3	PSC	M	77	No	-	No	Stent removal left hepatic duct	Left main duct	10.1	394
4	PSC	F	70	Yes	UC	No	Right hepatic duct stricture	Common Bile Duct, Common hepatic duct	0.3	159
5	PSC	F	34	Yes	UC	No	Follow up (? cystic duct malignancy)	Bile	-	207
6	PSC	M	56	Yes	UC	Yes	Elevated bilirubin	Common Bile Duct	6.6	552
7	PSC	F	60	Yes	CD	No	Follow up (Mass on MRI segment 4)	Bile	0.6	58
8	PSC	M	30	Yes	UC	No	Surveillance	Bile	1.7	210
9	PSC	M	63	Yes	UC	Yes	Biliary stent removal	Left main duct	2.8	408
10	PSC	M	60	No	-	Yes	Surveillance	Common hepatic duct	1.7	967
1	non-PSC	M	65	No	-	No	Bile leak, benign biliary stricture (Stent removal or exchange)	Common Bile Duct bifurcation	0.6	158
2	non-PSC	M	72	No	-	No	Benign biliary stricture with lab abnormalities	Distal common Bile Duct	-	279
3	non-PSC	M	80	No	-	No	Pancreatic and biliary stricture	Common Bile Duct	1.1	76
4	non-PSC	M	69	No	-	No	Chronic pancreatitis, stent change	Common Bile Duct	0.3	103
5	non-PSC	F	54	No	-	No	Abdominal pain with persistent elevation of liver enzymes.	Distal common Bile Duct	0.2	145
6	non-PSC	F	62	No	-	No	Follow-up of bile leak	Common bile duct, Bile: Cystic duct	0.7	126
7	non-PSC	M	66	No	-	No	Biliary stent removal	Distal common bile duct	1.1	137

Presence of cirrhosis was determined by one or more of the following diagnostic techniques: elastography, cross-sectional imaging with cirrhotic liver morphology, evidence of portal hypertension and/or liver biopsy.

## Data Availability

The single-cell RNA-seq datasets generated during the current study are available in the GEO database repository, GSE239283 (https://www.ncbi.nlm.nih.gov/geo/query/acc.cgi?acc=GSE239283). Please contact the corresponding author to request code for all analysis and any additional data from this study.

## Abbreviations

AASLD: American Association for the Study of Liver Disease
AIH: Autoimmune hepatitis
CADD: Combined Annotation Dependent Depletion score
CK7: Cytokeratin 7
CD: Crohn’s disease
3D: three-dimensional
DEG: differentially expressed genes
ECO: extrahepatic cholangiocyte organoids
ERC: endoscopic retrograde cholangiography
ES: Exome Sequencing
GEMs: Gel Beads-In-Emulsion
GS: genome sequencing
IBD: inflammatory bowel disease
IL-17: Interleukin 17
IL17RA-E: Interleukin 17 receptor A-E
MAIT: mucosal-associated invariant T-cells
Mdr2: Multi Drug Resistance 3
PD-L: programmed death ligand-1
PSC: Primary sclerosing cholangitis
scRNA-seq: single cell RNA sequencing
SOX9: SRY-Box Transcription Factor 9
UC: Ulcerative colitis
